# Evaluation of early warning signals for soil erosion using remote sensing indices in northeastern Iran

**DOI:** 10.1038/s41598-025-94926-x

**Published:** 2025-03-21

**Authors:** Abdolhossein Boali, Mohsen Hosseinalizadeh, Narges Kariminejad, Hamid Reza Asgari, Ali Mohamadian Behbahani, Babak Naimi, Vahid Shafaie, Majid Movahedi Rad

**Affiliations:** 1 Department of Arid Zone Management, Gorgan University of Agricultural Sciences and Natural Resources, Gorgan, Iran; 2 Department of Natural Resources and Environmental Engineering, College of Agriculture, Shiraz University, Shiraz, Iran; 3 Department of Biology, University of Utrecht, Padualaan 8, Utrecht, 3584 CH The Netherlands; 4 Department of Structural and Geotechnical Engineering, Széchenyi István University, Győr, 9026 Hungary

**Keywords:** Soil Erosion, Landsat satellite images, Warning, Indicators, ICONA model, Geomorphology, Environmental impact

## Abstract

Soil erosion represents a major challenge to natural resource conservation, causing land degradation, biodiversity loss, and diminished soil quality. This study explored the use of satellite imagery to evaluate the spatiotemporal risk of soil erosion in northeastern Iran. The ICONA model was applied to identify areas at severe erosion risk, while remote sensing indices (NDVI, NDSI, and TGSI) were employed to analyze erosion trends. NDVI is used to monitor vegetation health, NDSI detects soil salinity levels, and TGSI assesses topsoil grain size distribution, collectively providing critical insights into soil erosion risk in the study area. These indices, derived from the Google Earth Engine with a 30-meter spatial resolution and monthly temporal intervals (2003–2022), were assessed at 100 points, equally divided between eroded and non-eroded regions. Field data, including vegetation plots and soil profiles, were used to validate the remote sensing outputs. Early warning signals were analyzed through three statistical indices—autocorrelation coefficient, skewness, and standard deviation—using Kendall’s tau. Results revealed that 39.7% of the area falls under low erosion risk, 58.4% under medium risk, and 1.9% under severe risk. Significant breakpoints in NDSI and NDVI were identified in 2013, while TGSI showed no detectable change. Major shifts occurred near the Alagol, Almagol, and Ajigol wetlands and northern drylands. This study underscores the importance of integrating satellite data with field validation to improve soil management, protect biodiversity, and guide sustainable erosion mitigation strategies.

## Introduction

Soil erosion is a serious environmental challenge worldwide^1^. As soil erosion is a complex and multifactorial problem, it is necessary to perform detailed analyses and use data collected from field and laboratory experiments to predict and control this problem^2^. Various factors, such as scattered vegetation, loose surface soil, drought, and climate change, can increase the risk of soil erosion^3^. In recent years, water erosion assessment has emerged as one of the significant challenges in natural resource management and ecosystem sustainability. Among the commonly used empirical models for assessing water erosion are VIKOR, SWAT, RUSLE, and MPSIAC^4^. These models typically include coefficients and parameters that require extensive research or calibration based on local conditions^5^. Moreover, applying some of these models depends on access to baseline data and statistical information, which may not be readily available in many regions^6^. Therefore, remote sensing-based models have been proposed as a practical approach for assessing water erosion over large scales and across time. These models, particularly in areas with difficult access, allow us to quickly and cost-effectively gather the necessary spatial information^7^.

Additionally, remote sensing models enable the analysis of spatial and temporal changes in water erosion, contributing to a better understanding of the patterns and factors influencing this phenomenon^8^. In this context, various models have been developed for monitoring and assessing this issue, with one of the most effective being the ICONA model^9^. This model uses remote sensing data and spatial analyses to accurately and regionally evaluate water erosion^7^. Due to its ability to integrate spatial and satellite data, ICONA helps identify erosion-prone areas and predict their environmental impacts, thus aiding in optimal management decisions to mitigate erosion-related damage^5^. The ICONA model was selected due to its higher spatial and temporal resolution, which is especially advantageous for the remote sensing data used in this study. This allows for precise spatial mapping necessary for accurate soil erosion assessment. Unlike more complex models, ICONA provides acceptable accuracy with fewer indices, aligning well with our region’s conditions. These features make ICONA a suitable choice for soil erosion risk assessment, and the information obtained from this model can also be used as a basis for the development of early warning systems (EWS).

Empirical erosion assessment models can play a significant role in developing early warning systems for soil erosion. These models provide essential data for early warning systems by accurately evaluating erosion in various regions. Utilizing the results of these models alongside spatial data makes it possible to monitor and predict soil erosion changes continuously. This enables dynamic warnings to be issued for at-risk areas, allowing timely implementation of management measures. Early warning systems can help predict erosion events and facilitate timely response. Erosion prevention and soil conservation are essential for sustainable land management and ecosystem health. They play a major role in supporting these efforts by providing a valuable dataset for decision-makers. The early warning system (EWS) relies on various data sources, including meteorological, soil moisture measurements, topographical, and land-use data^10^. An effective EWS incorporates communication mechanisms to disseminate alerts and warnings to stakeholders, including farmers, land managers, and policymakers^11^.

Timely alerts are crucial for prompt action. Several EWS have used rainfall thresholds to trigger alerts^12^. The system warns against potential soil erosion risks when rainfall exceeds a certain threshold. Sensors and satellite-based technologies are commonly used for monitoring. Remote sensing is suitable for tracking land cover changes and identifying vulnerable areas. Recent advances in artificial intelligence and machine learning have enhanced the accuracy of soil erosion prediction models, allowing more precise EWS^13^. Using remote sensing indicators based on time-series datasets can help better understand areas at risk of soil erosion. For example, by examining temporal changes in vegetation, patterns can be identified and considered as the EWS of soil erosion. Increasing soil salinity and extreme changes in the soil grain distribution can also indicate soil erosion^14^. By identifying EWS, it is possible to be aware of the changes in indicators affecting soil erosion and take preventive measures to control and manage it. These measures include the cultivation of cover crops, water management, and development of bioengineering and conservation agriculture.

This study explores the efficacy of Landsat satellite imagery in assessing the spatiotemporal risks associated with soil erosion, focusing on the timing and localization of EWS that indicate reduced resilience and heightened erosion risks. The hypothesis posits that a time-series analysis of remote sensing data from various locations can enhance EWS in arid and semi-arid ecosystems. Gonbad Kavous County in northeastern Iran, which has experienced groundwater depletion and land-use changes over recent decades, is particularly vulnerable to land degradation and soil erosion, potentially leading to severe environmental impacts. By evaluating early warning signals through remote sensing indices, this research aims to identify and manage areas at high risk effectively, thereby improving soil management practices and conserving natural resources in Gonbad Kavous. This could lay the groundwork for crafting sustainable policies to mitigate soil erosion.

## Materials and methods

### Study region

**Fig. 1:**
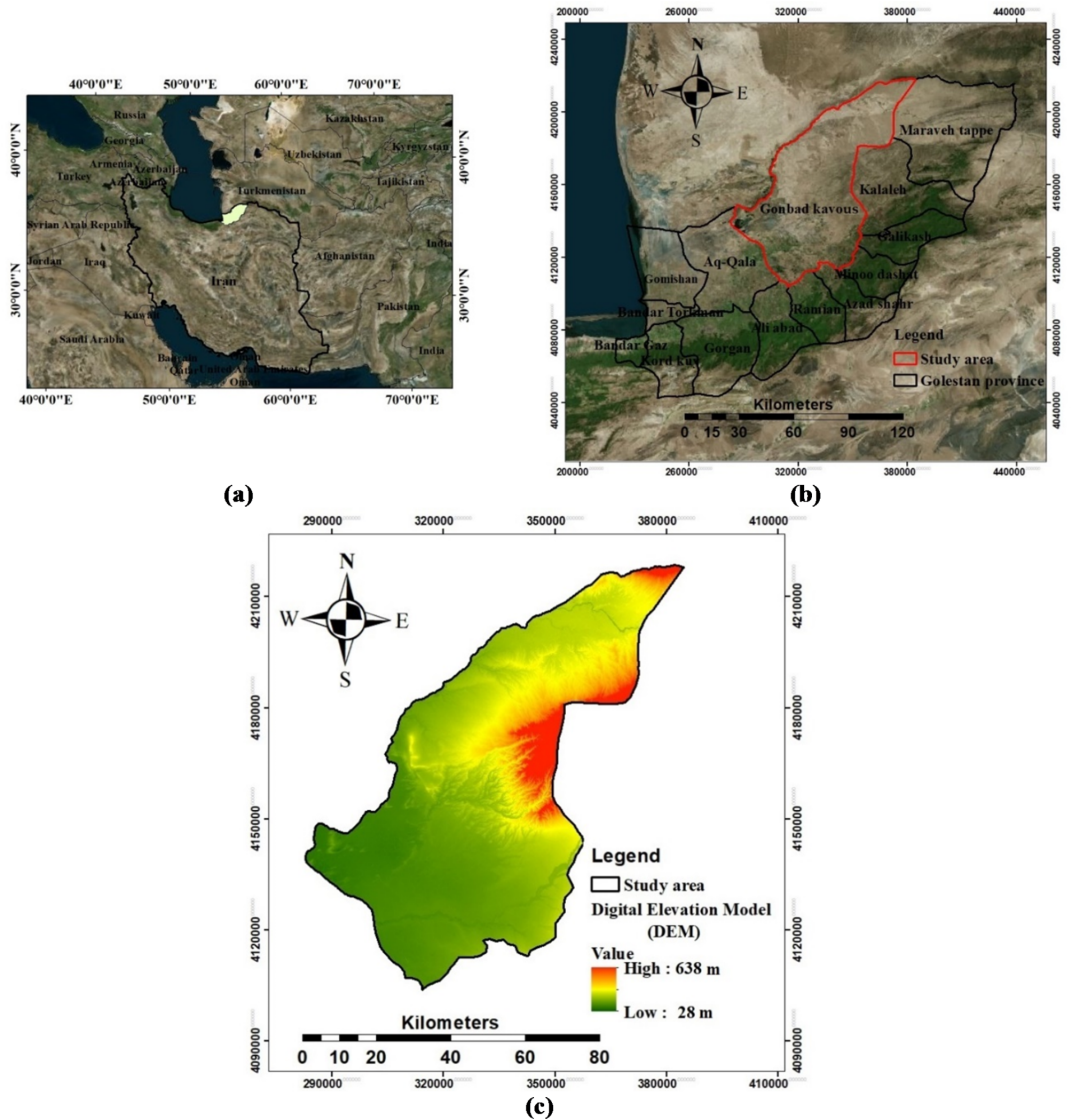
**a**) The location of the study region in Iran, **b**) Google Earth images, and **c**) Digital elevation model.

The study focuses on Gonbad Kavous County, which spans an area of 4,995 km^2^ and is situated within Golestan Province in northeastern Iran. This area is located at an eastern longitude of 54 °and 31 min, maximum of 55 °and 39 min, northern latitude of 37 °and 3 min, and maximum of 38 °and 6 min. The region exhibits significant elevation variation, with the easternmost areas reaching an altitude of approximately 638 m above sea level, and the southwestern areas descending to around 28 m. Most of the area is used for pastures covered by floods and wind sediment. Over the two-decade period from 2001 to 2021, the study region recorded an average annual rainfall of 388 mm and a mean temperature of 24.05 °C. This region faces soil erosion problems owing to special geological and climatic features, such as steep hills in the west, sparsely covered and poor lands in the north, and heavy seasonal rains (Fig. 1).

### Research data

NDVI (Normalized Difference Vegetation Index), NDSI (Normalized Difference Salinity Index), and TGSI (Topsoil Grain Size Index) are key remote sensing indices used in environmental studies. NDVI, ranging from − 1 to + 1, assesses vegetation health and density, with higher values indicating denser, healthier vegetation. NDSI, typically ranging from − 1 to + 1, evaluates soil salinity, where higher positive values suggest higher salinity levels. TGSI, usually scaled between − 0.5 and 0.2, analyzes topsoil particle size distribution, with higher values indicating larger grain sizes like sand (Table 1). These indices provide crucial information about vegetation cover, soil characteristics, and surface conditions, enabling comprehensive analysis of ecosystem health and land degradation processes.

Landsat Tier 1 surface reflectance images, selected for analysis, underwent atmospheric and geometric corrections, along with cross-calibration between sensors, prior to use. Generated through Google Earth Engine, these indices utilize a 30-meter resolution to allow for high precision in mapping erosion risk. Monthly time intervals spanning from 2003 to 2022 enhance the temporal aspect and capture seasonal and interannual changes in soil erosion patterns. Field data was utilized to validate the remote sensing indicators. In July 2022, soil samples were collected from the western regions near Alagal Lagoon to assess salinity and soil texture. Furthermore, vegetation assessment was conducted by measuring one-square-meter plots along nine 100-meter transects within the area, with vegetation coverage in each plot determined through expert evaluation.

All analytical and image-processing tasks were carried out utilizing R (version 4.3.2, 2023)^15^ statistical software in conjunction with the Google Earth Engine platform^16^. Additional packages utilized in this study include the raster package for analyzing various raster maps^17^, the rts package for time-series analysis^18^, and the BFAST package for detecting breaks in seasonal/trend patterns^19^. The “earlywarnings” and “spatialwarnings” packages were also employed for studying ecosystem EWS^20,21^.

**Table 1:** List of the datasets applied in the present study.

Name	Formula	Range	product	Ground surface data	Spatial resolution and sensor	year	References
TGSI	R – B / R + B + G	−1 to + 1	Landsat (5–7–8)	Soil surface profile	30 M	2003–2022	^22,23^
NDSI	R – NIR / R + NIR						^24^
NDVI	NIR – R / NIR + R			Vegetation percentage in plots			^22,25^

### The ICONA model for water erosion assessment

**Fig. 2:**
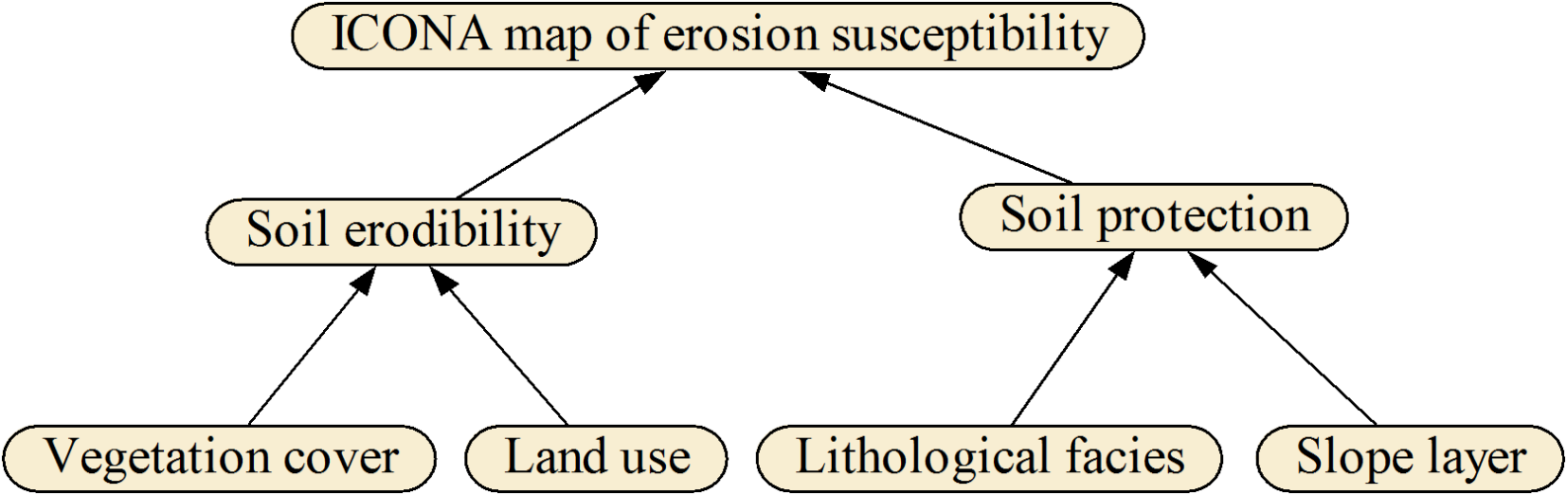
The methodological architecture of the ICONA model.

The Spanish Nature Protection Association introduced the ICONA model for evaluating water erosion. The efficiency of this model has been proven in various regions worldwide^26^. Due to its remote sensing-based approach, this model has shown superiority over standard empirical models in this field of research^5^. The ICONA model is a good choice compared to other models, such as RUSLE, SWAT, etc., due to its simplicity of implementation and the ability to use remote sensing data. Although the mentioned empirical models have high accuracy in calculating erosion rates, they can create limitations due to the need for large amounts of data and complexity in implementation. Figure 2 illustrates the steps involved in implementing the ICONA model. In this model, the soil erodibility map is generated by superimposing the layers of slope and geology, a process facilitated by the use of ArcGIS 10.7.1^27^. Conversely, the soil protection map is created by superimposing the land use and vegetation cover layers. Ultimately, the soil erosion susceptibility map, as defined by the ICONA model, results from the integration of the soil erodibility and soil protection maps.

### Soil erodibility mapping

To develop a soil erodibility map, the slope and geological maps are initially prepared. The slope map for the area is extracted from a Digital Elevation Model (DEM) map, which is sourced from the United States Geological Survey (USGS) website. A digitized geological map at a 1:100,000 scale will be employed to classify the rock surface layers within the area, categorizing geological formations into five groups according to their resistance to weathering, as outlined in Table 2. Subsequently, by overlaying the slope and geological maps, the soil erodibility layer will be classified into five categories: very low (EN), low (EB), moderate (EM), high (EA), and very high (EX) (Table 3)^9^. As indicated in Table 3, when both geology and slope are categorized in class one, the classification for soil erosion susceptibility is also assigned to class one.

**Table 2:** Lithofacies and slope classes.

Class	Lithofacies	Slope
1	Unweathered dense rocks, well-cemented conglomerates or soils, crust formations, and hardpans, including massive limestone, rocky soils with high stone content, and igneous or volcanic rocks.	Flat to gentle (0–3%)
2	Fractured and/or moderately weathered cohesive rocks or soils.	Moderate (%3–12%)
3	Sedimentary rocks with low to moderate compaction, such as slate, schists, and compacted marls.	Steep (%12–20%)
4	Soft, low-strength rocks or those that are heavily or deeply weathered, including marl, gypsum, and clayey slates.	Very steep (%20–35%)
5	Unconsolidated, non-cohesive sediments, soils, and detrital materials.	Extremely steep (> 35%)

**Table 3:** Calculation of soil erodibility classes based on slope and lithofacies classes.

Slope Class	Lithofacies class				
	1	2	3	4	5
1	1 (EN)	1 (EN)	1 (EN)	1(EN)	2 (EB)
2	1 (EN)	1 (EN)	2 (EB)	3 (EM)	3 (EM)
3	2 (EB)	2(EB)	3 (EM)	4 (EA)	4 (EA)
4	3 (EM)	3 (EM)	4(EA)	5 (EX)	5 (EX)
5	4 (EA)	4 (EA)	5 (EX)	5 (EX)	5 (EX)

### Soil conservation mapping

The soil conservation map is derived by integrating the land use and vegetation cover maps. To generate the land use map within the Google Earth Engine platform, the study area is first delineated, and its geographical boundaries are precisely defined. Landsat satellite imagery undergoes rigorous atmospheric, geometric, and radiometric corrections to ensure data reliability. Field observations and existing datasets are then utilized to select training samples, identifying representative points for different land use categories across the region. Subsequently, land use classification is executed using advanced machine learning techniques, specifically Support Vector Machine (SVM) and Random Forest (RF) algorithms. The accuracy of the generated maps will be evaluated using overall accuracy and Kappa coefficient metrics to ensure that the most accurate map is incorporated into the modeling process. The NDVI index was used to create the vegetation cover map. This index was calculated in Google Earth Engine using Landsat 8 images and the relationships between their bands^28,29^. The classification of the vegetation cover map is presented in Table 4.

The soil conservation status is classified into five distinct categories: very high conservation (MA), high conservation (A), moderate conservation (M), low conservation (B), and very low conservation (MB), as outlined in Table 5^9^. As shown in Table 5, if the land use is classified as rainfed agriculture and the vegetation cover percentage is between 0 and 25%, the soil conservation class will be classified as class one.

**Table 4:** Land use and vegetation cover classes.

Class	Land use	Vegetation cover
1	Dry Farming	0–25%
2	Irrigated Farming	25–50%
3	Sand Areas	50–75%
4	Rangeland	> 75%
5	Bare Land	-
6	Rock Outcrops	-

**Table 5:** Soil protection index classes.

Land use	Vegetation cover			
	0–25%	25–50%	50–75%	> 75%
Dry Farming	MB	MB	B	B
Irrigated Farming	MB	MB	B	M
Sand Areas	M	M	A	MA
Rangeland	MB	B	M	A
Bare Land	MB	M	A	MA
Rock Outcrops	MA	MA	MA	MA

The erosion risk map, derived using the ICONA model, is created by integrating the soil erodibility and conservation maps. The erosion risk is then categorized into five distinct classes: shallow (Class 1), low (Class 2), moderate (Class 3), high (Class 4), and very high (Class 5), as presented in Table 6^7^.

**Table 6:** Erosion hazard classes.

Soil protection	Soil erodibility				
	EN	EB	EM	EA	EX
MA	1	1	1	2	2
A	1	1	2	3	4
M	1	2	3	4	4
B	2	3	3	5	5
MB	2	3	4	5	5

### Validation of the water erosion risk map

The accuracy of the model results was assessed using the erosion risk map derived from the ICONA model. A total of 100 points were systematically and randomly selected for evaluation across different water erosion classes. These points were analyzed through observational and visual assessments, and the results were statistically compared against expert judgments. Validation was conducted using the non-parametric Mann-Whitney U test. This test is a suitable method for comparing two independent groups and allows us to identify significant differences between predicted values ​​and expert judgments. In this test, values range from − 1 to 1: a value of 1 indicates that the model’s prediction exceeds the expert judgment and observed ground truth; a value of −1 signifies that the prediction falls below the ground truth; and a value of 0 denotes complete agreement between the model, expert judgment, and ground truth.

### Investigation of breaking points in the time series of remote sensing indicators

After identifying areas with severe erosion, the necessary investigations were carried out to provide a soil erosion warning system. By reviewing comprehensive sources, three remote sensing indices NDVI, NDSI, and TGSI were used to investigate soil erosion changes in the framework of the warning system. Although a review of extensive sources has reported a history of sudden changes in the western areas of the study area (environmental department reports), we used the BFAST (breaks for additive trend and seasonal) package to determine the time of sudden changes (breakpoint) in each of the remote sensing indicators affecting soil erosion in the study area^19^. The breaking point refers to the largest sudden change in the time series of indicators between 2003 and 2022, indicating soil erosion. To check the occurrence of the breaking point, a monthly map of each of the investigated indicators was prepared using Google Earth Engine. In continuation of the time series, the indices were divided into trend and seasonal components. The trend component captures long-term variations, while the seasonal component reflects periodic and short-term fluctuations in the data. Subsequently, using the results of the ICONA model, change trends were analyzed employing the BFAST method at 100 selected points—50 located in areas classified as experiencing severe soil erosion and 50 in areas classified as low erosion^19^.

This analysis considers only the largest breakpoint for each time series. Following the identification of the breakpoint, the analysis focused on the period from 2003 up to two years prior to the detected abrupt change. The inclusion of a two-year period aligns with the methodology employed in previous research studies^30,31^.

### Methods of detecting time-spatial EWS

We applied Kedall’s tie test between each remote-sensing index and its time series to check for warning signals. Kedall’s tie is a non-parametric test used to measure the relationship between two measured quantities, which vary between 1 and − 1. Larger Kendall values indicate stronger trends. Kendall’s tau was additionally utilized to evaluate the significance of changes in the statistical indices, including the autocorrelation coefficient, skewness, and standard deviation.

These indicators were estimated using an Early Warning package in the R (version 4.3.2, 2023) software environment^21^. These findings suggest that as the system approaches its peak point gradually, there will be a notable increase in the autocorrelation coefficient well ahead of the main transition^20^. Autocorrelation enhancement indicates that the system’s state becomes more alike in successive observations^20^, and it can be viewed as the most basic method to assess critical implications^21^. Auto-correlations can be determined by considering a specific time delay. Previous studies on leading indicators typically utilize one-step time lags or log-1 autocorrelation. Hence, it can be computed using the following formula.

4 $$\:{P}_{1}=\:\frac{E\left[{\langle}{Z}_{t}-\:\mu\:{\rangle}{\langle}{Z}_{t+1}-\:\mu\:{\rangle}\right]}{\delta{}_{z}^{2}}$$

The expected value E, mean µ, and variance δz of the variable zt are connected. If the system slowly returns to a steady state, it can lead to wider fluctuations around that state, ultimately increasing the variance before a transition^32^. Variance, representing the second moment about the mean in a spatial distribution, can be expressed in terms of the standard deviation as follows:

5 $$\:SD=\:\sqrt{\frac{1}{n-1}\:\sum\:_{t=1}^{n}{{\langle}{Z}_{t}-\:\mu\:{\rangle}}^{2}}$$

Additionally, an increase in the asymmetry of fluctuations may arise due to an unstable equilibrium near the peak point, independent of changes in the autocorrelation coefficient and variance. The standardized third moment’s skewness can be calculated near the distribution’s mean using the equation below:

6 $$\:SK=\:\frac{\frac{1}{n}\sum\:_{t=1}^{n}{\left({Z}_{t}-\mu\:\right)}^{3}}{\sqrt{\frac{1}{n}}\sum\:_{t=1}^{n}{\left({Z}_{t}-\mu\:\right)}^{2}}$$

Prior to analyzing the time series of the remote sensing indicators, the dataset was detrended and filtered to eliminate non-stationary effects and ensure a stable time series for analysis. Commonly used data detrending methods include the Gaussian filter, LOSS fitting, linear detrending, and first difference. A moving window between 25% and 75% of the time-series length was used to examine the changes. To identify the most effective data-detrending methods for detecting changes in the indicators, a sensitivity analysis was conducted using various moving window sizes ranging from 25 to 75% of the time series length. This analysis aimed to determine the optimal moving window size for the dataset.

Using Kedall’s test, the strength of spatial changes in each considered environmental indicator was investigated at the regional level. Therefore, areas with the most significant changes in vegetation cover, soil salinity, and surface particle size were identified as those at risk of erosion.

## Results

### Erosion hazard map

**Fig. 3:**
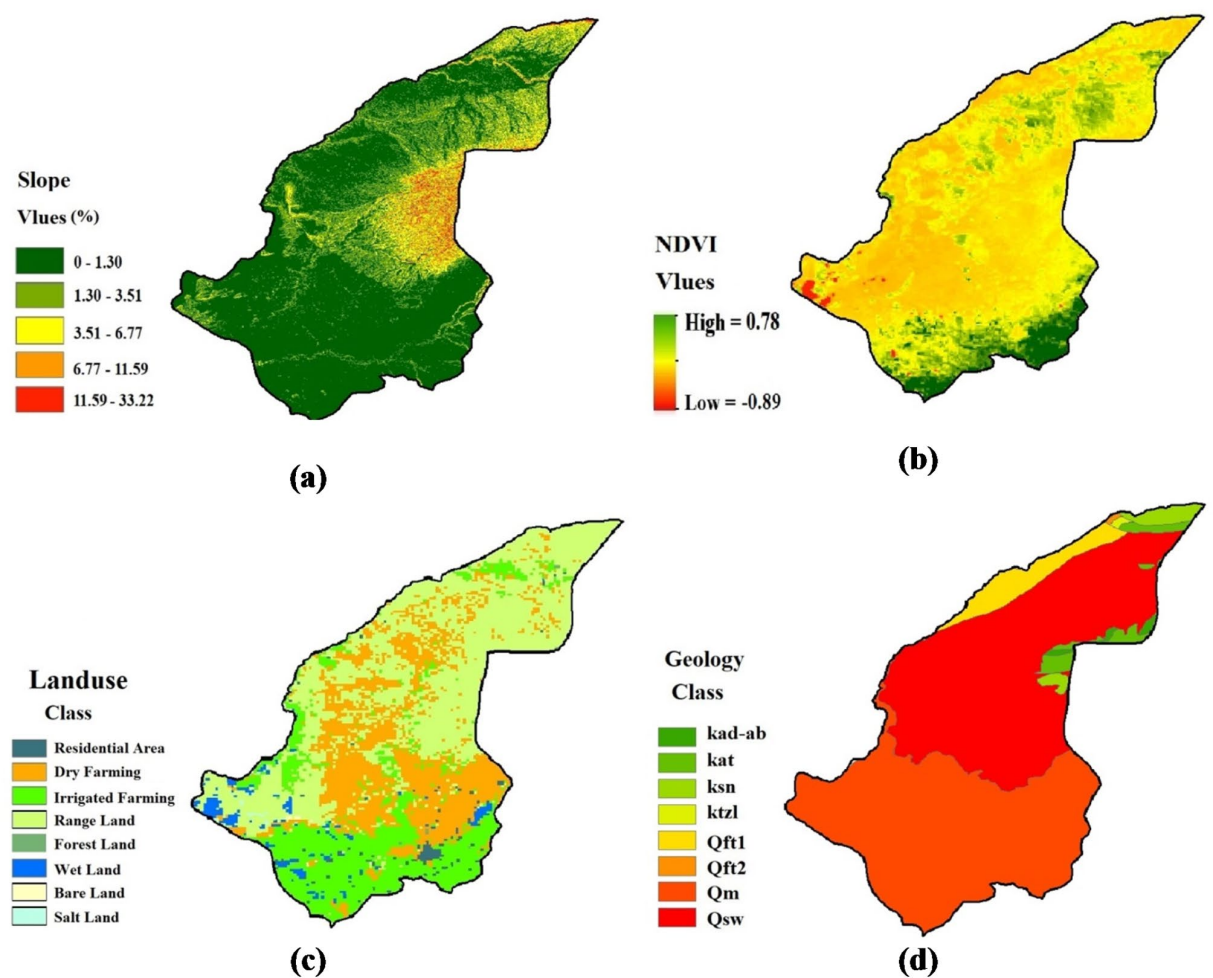
Input maps of the ICONA model: (**a**) Slope map, (**b**) Vegetation cover map, (**c**) Land use map, (**d**) Geology map.

Figure 3 presents the input maps of the ICONA model. The vegetation cover map indicates dense vegetation in the southern areas and sparse vegetation in the northern regions. The steepest slope (33%) was observed in the area’s southeast. Rainfed and irrigated agricultural lands are the dominant land uses in the region, and Quaternary formations are the predominant geological formations in the area.

**Fig. 4:**
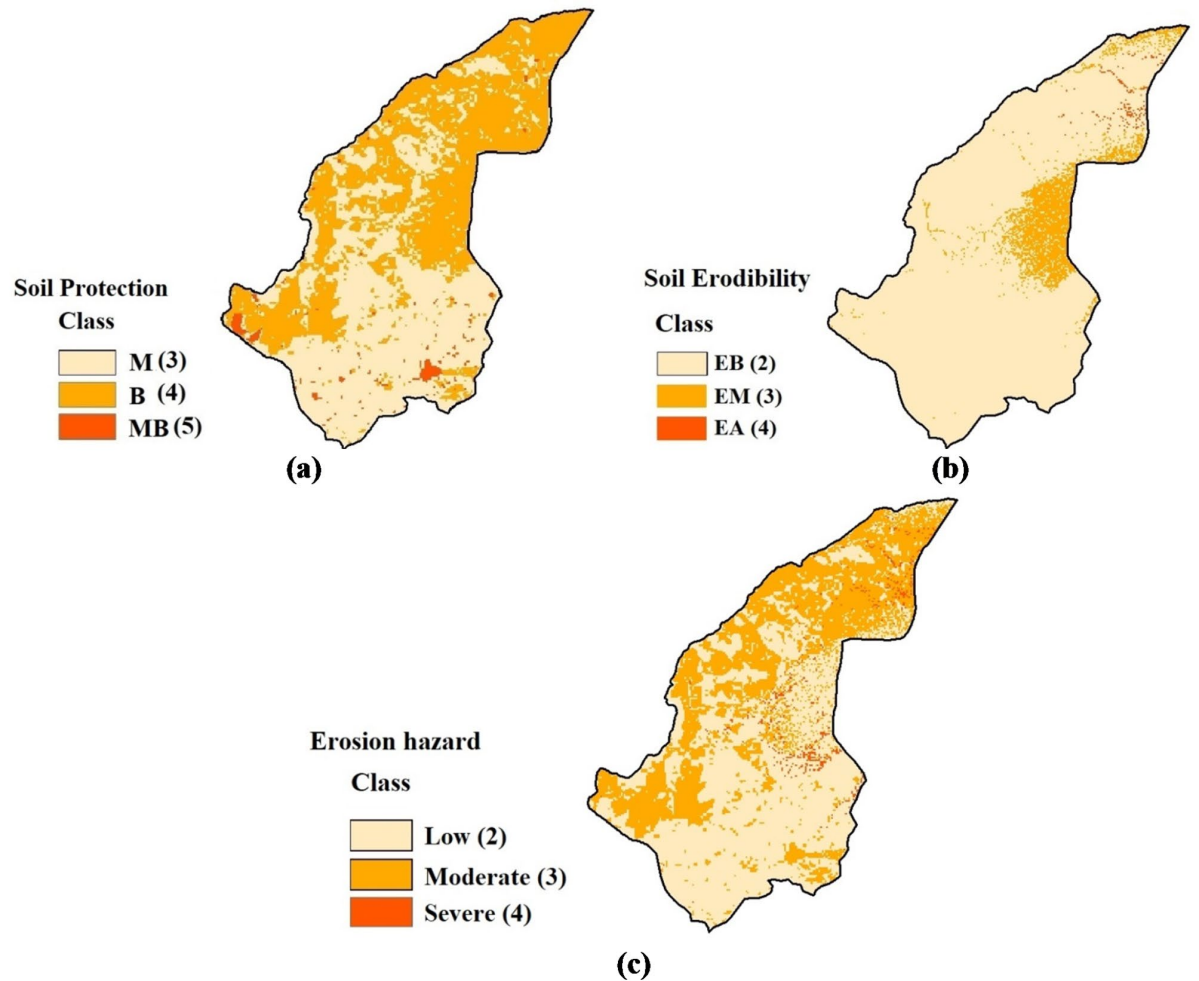
Soil erosion risk modeling based on the ICONA model: **a**) soil protection map, **b**) soil erodibility map, **c**) soil erosion hazard map.

The soil conservation map revealed that 50.83% of the region is classified as having moderate conservation status, 47.33% as low conservation, and 1.82% as very low conservation. Notably, the northern areas of the study region are characterized by poor soil conservation. Analysis of the soil erodibility map indicated that 89.6% of the area falls within the low erodibility class, 9.8% within the moderate class, and 0.6% within the high erodibility class, with the eastern parts exhibiting the most severe erodibility. Using the ICONA model, an erosion hazard map was generated by overlaying the soil erodibility and conservation maps (Fig. 4). This map revealed that 39.7% of the area falls within the low erosion risk category, 58.4% within the moderate risk category, and 1.9% within the severe risk category. The final classification of the erosion hazard map underscores significant erosion risks in the eastern, western, and northern parts of the region.

The statistical analysis indicates that the ICONA model demonstrates a aproparate correspondence and correlation with ground data.The P-value of 0.68 indicates a moderate but not perfect correlation between model results and ground truth data. In soil erosion studies, such a P-value suggests a reasonable level of model reliability, though further refinement could enhance accuracy.

### Time analysis of the EWS

**Fig. 5:**
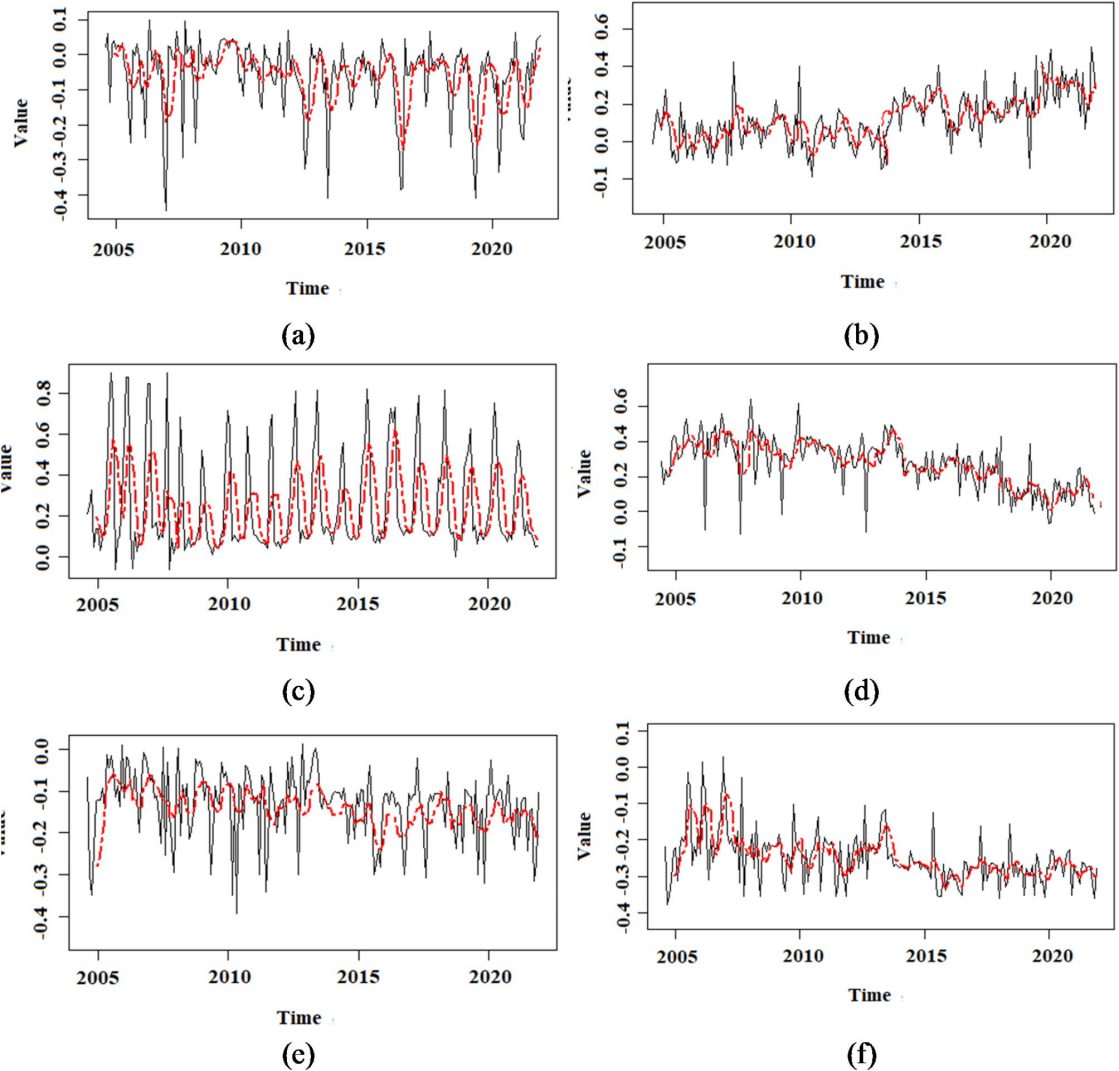
The sequential data of three remote sensing indices: Normalized Difference Salinity Index (NDSI), Normalized Difference Vegetation Index (NDVI), and Topsoil Grain Size Index (TGSI). These indices were utilized as variables in both eroded and non-eroded regions. The NDSI, NDVI, and TGSI indexes represent areas without erosion (a-f) and areas with erosion, respectively. Additionally, a red line indicates the moving average with a time step of 3 in the time series of the indicators.

The current research employed NDVI, NDSI, and TGSI remote sensing indices to examine soil erosion changes. Figure 5 shows the trend of changes in these indicators in eroded and non-eroded areas. The most prominent breaking point in NDSI and NDVI environmental indicators was observed in 2013. However, TGSI did not show any detectable breaking point (Fig. 5). The BFAST model demonstrated a confidence level of 95%. After the identified breakpoint in the eroded regions, a notable increase in NDSI was observed, while NDVI exhibited a corresponding decline, reflecting significant environmental changes. An increasing trend in NDSI suggests rising soil salinity in eroded areas, which may further exacerbate erosion risk. Meanwhile, a decline in NDVI reflects vegetation loss, amplifying the vulnerability of the soil to further erosion. In areas without erosion, the trend of changes over the entire period (2003–2022) was uniform (Fig. 5).

**Fig. 6:**
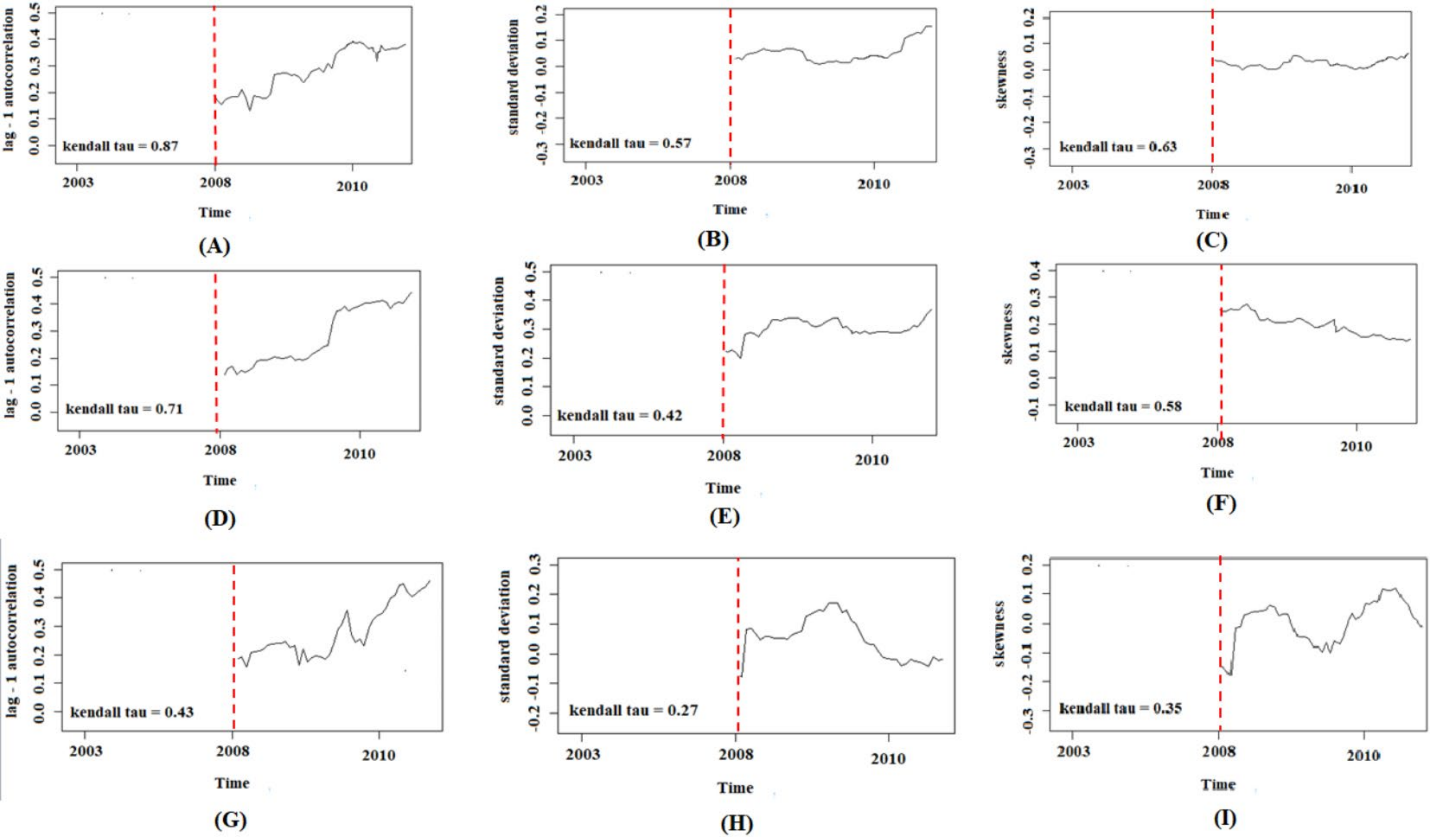
The auto-correlation coefficient, standard deviation, and skewness in graphs** A**, **B**, and **C** for the NDVI time series, graphs **D**, **E**, and F for the NDSI time series, and graphs **G**, **H**, and **I** for the TGSI time-series. Each graph shows the auto-correlation values on the y-axis and time (years) on the x-axis, ranging from 2003 to two years before the sudden change identified in each pixel. The dotted red lines indicate the rolling window size used to compute the auto-correlation coefficient, standard deviation, and skewness.

The temporal trends of EWS in eroded areas were systematically examined. Analysis of the NDVI time series revealed a significant upward trend in the autocorrelation coefficient, standard deviation, and skewness, with Kendall’s tau values of 0.87, 0.57, and 0.63, respectively, across the majority of pixels (Fig. 6). For the NDSI time series, an increasing trend was observed in the autocorrelation coefficient and standard deviation, while skewness exhibited a decreasing trend, with Kendall’s tau values of 0.71, 0.42, and 0.58, respectively. Similarly, the TGSI time series demonstrated a positive trend in the autocorrelation coefficient and a negative trend in both standard deviation and skewness, with Kendall’s tau values of 0.43, 0.27, and 0.35, respectively. These trends were found to be statistically significant for all three indices, even when analyzed under varying combinations of rolling window sizes and filter bandwidths (Fig. 6).

These results indicate significant changes in the ecosystem structure of the study area. The simultaneous increase in NDVI statistical indicators may suggest a decrease in ecosystem resilience. At the same time, the differences in observed trends for NDSI and TGSI reflect the complexity of processes involved in soil and vegetation cover changes. These alterations may indicate the ecosystem’s movement towards a critical point, where the system might abruptly transition to a new state. Furthermore, the variation in trends observed across different indices underscores the importance of employing multiple approaches in assessing ecosystem health and predicting future changes. This multi-faceted analysis provides a more comprehensive understanding of the ecosystem dynamics and potential tipping points, enabling more informed decision-making in ecosystem management and conservation efforts.

### Investigating the trend of spatial changes of soil erosion based on the changes in Kendall Tai environmental indicators

**Fig. 7:**
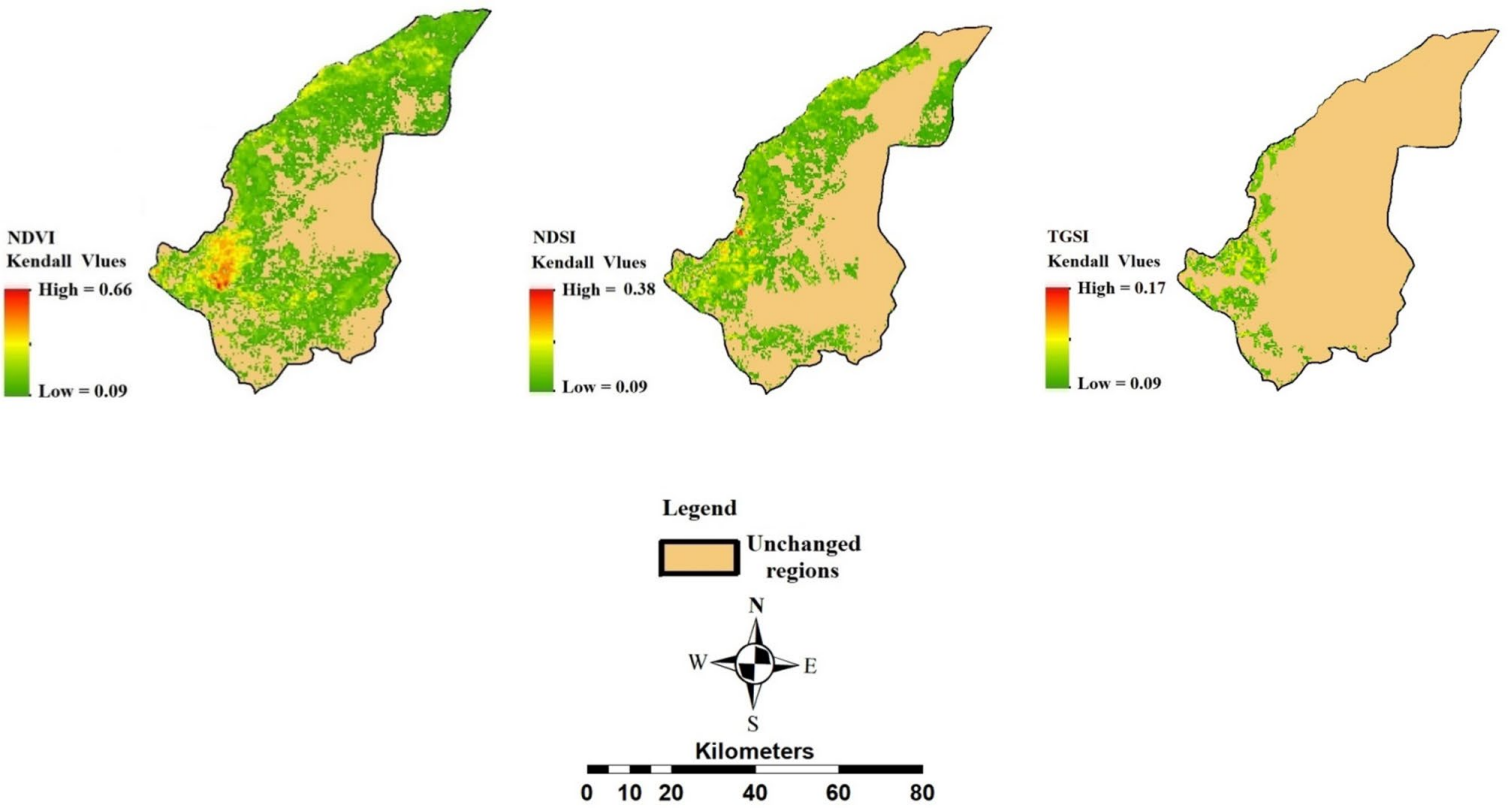
Examining the trend of spatial changes of environmental indicators based on Kendall-Thai changes.

Examining the trend of spatial changes in environmental indicators showed that the most significant changes occurred in the western parts of the region. According to Kendall’s statistical test, the powers of change for the NDVI, NDSI, and TGSI were 0.66, 0.38, and 0.17, respectively. Kendall’s tau values greater than 0.5 indicate strong autocorrelation in the time series data, suggesting that observed changes in NDVI and NDSI are likely indicative of genuine trends in vegetation and soil conditions over time. According to the NDVI, most changes occurred in the areas around the Alagol, Almagol, and Ajigol wetlands, and to some extent in the northern hillslopes of the region (Fig. 7). In terms of changes in the NDSI, the most significant changes in soil salinity were observed in the rainfed fields north of Ajigol Lake. The trend of changes in TGSI in the region was limited to the western areas and around the wetlands.

## Discussion

Soil erosion poses a serious threat to various ecosystems, particularly in semi-arid regions, and can significantly impact agricultural productivity and biodiversity. This study introduces a new method for assessing EWS of soil erosion using analytical tools and environmental data. Analyzing soil test results and environmental indicators can help detect potential soil erosion risks before they manifest. Based on the ICONA model, a soil erosion hazard map was produced. The model indicated that 39.7% of the area falls into the low erosion risk category, 58.4% into the moderate risk category, and 1.9% into the severe erosion risk category, primarily located in the eastern, western, and northern parts of the region. The ICONA model’s utility in assessing water erosion risk is supported by previous studies^7^. The ICONA model, while useful for assessing soil erosion risk, has limitations. Its accuracy depends heavily on the availability of high-quality input data, which may be a challenge in some regions like Gonbad Kavous. Furthermore, the model simplifies complex erosion processes and ideally requires local calibration to ensure reliable results^7^. Subsequently, remote sensing indices were analyzed within areas classified by ICONA to determine how these indices behave in regions identified by the model as having high or low erosion. This approach enables the analysis of remote sensing indices about the spatial distribution of erosion risk, as defined by the ICONA model.

The study focuses on monitoring changes in vegetation indices, soil salinity levels, and the distribution of soil particles over time and space to generate effective EWS for soil erosion prevention^3^. The results of investigating the EWS between 2003 and the two years before the sudden changes leading to soil erosion (2013) were investigated using three statistical indicators in the western eroded areas and areas without soil erosion in the northern and southern regions. The autocorrelation coefficient’s statistical index correctly showed a changing trend in most of the examined pixels. This index not only focuses on the average and average data changes but also considers the time pattern of changes^32^, it was better than the other statistical indicators used (standard deviation and skewness). The results showed significant changes in the autocorrelation coefficients of the environmental indices NDVI and NDSI in the western eroded areas. The significant increase in NDVI and NDSI before 2013 indicates real signals for soil erosion in the region. The trend of changes in TGSI based on the statistical indicators investigated in different parts of the region was irregular and showed no signs of erosion risk.The marked increase in NDSI post-2013 suggests a rise in soil salinity in eroded areas, which further exacerbates erosion risk. Simultaneously, the decline in NDVI indicates vegetation degradation, increasing the soil’s vulnerability to further erosion^10^. The observed increase in the autocorrelation coefficient is often associated with greater variability (e.g., standard deviation) and asymmetry (e.g., skewness). However, this relationship is not always definitive. As discussed by^21^, an increase in the autocorrelation coefficient without corresponding increases in variability and asymmetry might indicate potential ecosystem recovery, suggesting that the system is stabilizing despite past disturbances. Conversely, a simultaneous rise in all indicators could signify reduced resilience and a trend toward instability or collapse of the ecosystem^33^. In general, the better performance of NDVI and NDSI compared to other indices can be attributed to various reasons, including greater sensitivity to environmental changes, higher accuracy and interpretability^34^ and a remarkable increase in the autocorrelation coefficient (Fig. Kendall = 0.87) before 2013 and a strong considerable decline (Kendall = 0.79) after that time could indicate a critical transition, with a peak at the time of trend change (2013)^19^. The trend of positive changes before a sudden change indicates a slowing down of the ecosystem, and acts as an early warning signal for a critical transition. The trend of negative changes after the peak point also indicates a new stabilized state of the ecosystem after the critical transition^20^. These observed patterns and relationships among statistical indices and remote sensing data align with findings from previous research, further validating the use of these methods for identifying critical transitions and assessing ecosystem resilience^11^.

Temporal and spatial data analysis approaches have advantages and limitations in early warning-signal detection. Temporal examination of data can sometimes produce false alarms, particularly when dealing with large accumulations of temporal data. However, the spatial data analysis approach provided detailed information regarding the distribution of the changes. Nevertheless, its effectiveness is constrained by its sensitivity to pre-processing procedures^31^. Research has demonstrated that the ability to detect EWS is heavily reliant on the quality of satellite image resolution^35^. Higher-resolution images allow for more precise signals to be captured. Utilizing satellite images with superior spatial resolution may assist in improving the computational process^32^. Landsat satellite images have a suitable resolution, which enables the detection of accurate changes in different areas. As powerful and appropriate data sources, these images provide essential information for monitoring and analyzing warning signals, which can be useful for predicting and effectively managing environmental risks and threats. Although Landsat imagery is usually effective, we found it challenging to detect minimal changes in vegetation, especially in certain areas. Because of this, we might have underestimated early warning signals in these areas. Future studies should look at how the scale of change affects detection and try using techniques that automatically adjust the sensitivity to better identify these subtle changes.

The application of remote sensing indices such as NDVI, NDSI, and TGSI in this study aligns with previous research that has utilized these indices to monitor vegetation health, soil salinity, and topsoil characteristics^36–38^. For instance, the use of NDVI has been instrumental in assessing vegetation cover and its correlation with soil erosion rates, as demonstrated in prior studies that identified significant relationships between vegetation dynamics and soil stability^36,38^. Similarly, NDSI has been applied to detect soil salinity levels, which are critical in understanding erosion dynamics and soil degradation processes^37^. The integration of multiple remote sensing indices provides a comprehensive approach to evaluating soil erosion risk, reinforcing findings from previous studies that emphasized the importance of spectral indices in assessing landscape vulnerability^38,39^.

Furthermore, the identification of significant breakpoints in NDVI and NDSI around 2013 corresponds with studies that have observed spatiotemporal variability in land degradation patterns, often attributed to climatic fluctuations and anthropogenic activities^39,40^. The incorporation of statistical analyses, such as autocorrelation coefficients, skewness, and standard deviation, to detect early warning signals of soil erosion, complements methodologies employed in prior research, which demonstrated that geostatistical modeling and time-series analysis are essential for capturing soil degradation trends^37,40^. This study’s integration of satellite data with field validation underscores the importance of combining remote sensing with ground-truth data to improve soil management and guide sustainable erosion mitigation strategies^40^. These findings contribute to the ongoing efforts to develop proactive monitoring systems for land conservation and environmental sustainability.

## Conclusion

Soil erosion has been extensively examined as a hydropedo-geomorphological process that substantially contributes to slope and catchment hydrology, soil loss, and landscape evolution. Nevertheless, it is imperative to recognize that soil erosion can exert a substantial, temporally variable impact on the environment and society. The design and operationalization of dedicated early warning systems for soil erosion remain limited in their global implementation. Further research is imperative to deepen our understanding of these phenomena and enhance their predictive capacity. Priority consideration should be given to natural hazards precipitated by soil erosion, such as landslides, land degradation, subsidence, flooding, and sediment-related predicaments. Enhancing the prevention and control measures in regions prone to erosion requires a more profound understanding of the interrelationships between these phenomena.

This study showed that using the analytical and effective approach presented in this study can significantly help identify the EWS of soil erosion. Recognizing the profound implications of soil erosion for agriculture, environmental sustainability, and regional economic stability, it is imperative to prioritize further research and implement practical measures aimed at improving soil quality, safeguarding water resources, and preserving biodiversity. Given the complexity and multifactorial nature of soil erosion, conducting comprehensive analyses supported by field and laboratory data is essential for accurately predicting and effectively mitigating this issue. This study showed that remotely sensed indices such as NDVI, NDSI, and TGSI can act as early warning signals for reduced ecosystem resilience, indicating greater vulnerability to soil erosion. Monitoring these indices allows for proactive management of soil erosion by identifying areas where ecosystems are declining. The findings of this study can contribute to the development of targeted erosion control strategies in Gonbad Kavous County. The results may inform vegetation restoration initiatives and soil salinity management programs aimed at mitigating soil erosion risks. To ensure effective implementation, local authorities should prioritize allocating resources for these strategies, fostering collaboration between researchers and practitioners, and establishing monitoring programs to assess the long-term impact of these interventions. Future research should incorporate high-resolution spatial data and drone imagery to analyze microclimatic effects on soil erosion and EWS detection at a finer scale. Furthermore, investigating the human factors contributing to soil erosion in Gonbad Kavous County, such as land management practices and socioeconomic drivers, would provide a more holistic understanding of the problem and inform more effective intervention strategies.

## Data Availability

The datasets used and/or analysed during the current study available from the corresponding author on reasonable request.
